# How Long? How Many? How Much? Evidence of Convergent Validity Among Thin-Slice Behavioral Coding Metrics

**DOI:** 10.1007/s10919-025-00489-w

**Published:** 2025-07-26

**Authors:** Nora A. Murphy, Mollie A. Ruben, Morgan Stosic, Laetitia A. Renier, Katja Schlegel, Judith A. Hall, Marianne Schmid Mast, Brett Marroquín

**Affiliations:** 1 Loyola Marymount University, Los Angeles, USA; 2 University of Rhode Island, Kingston, USA; 3 University of Maine, Orono, USA; 4 KBR, Behavioral Health and Performance Laboratory, Biomedical Research and Environmental Sciences Division, Human Health and Performance Directorate, NASA Johnson Space Center, NASA Johnson Space Center, USA; 5 University of Lausanne, Lausanne, Switzerland; 6 University of Bern, Bern, Switzerland; 7 Northeastern University, Boston, USA

**Keywords:** Behavioral Coding, Thin Slices, Convergent Validity, Gaze, Nod, Smile, Gesture

## Abstract

Despite the ubiquity of thin-slice coding for behavioral measurement, there exists relatively little systematic research into the convergent validity of thin-slice coding metrics for nonverbal behaviors when using human coders. This study utilized five previous datasets to measure four commonly-measured nonverbal behaviors (gaze, gestures, nods, smiles) using three different coding metrics (duration, frequency, rating) coded in 2 or 3 min slices. Convergent validity was measured by comparing a given behavior coded with at least two different metrics. Meta-analytic assessments across studies, behaviors, and metrics indicated strong convergent validity for various metrics for each behavior. Results provide confidence to researchers on the validity of using these thin-slice coding metrics for nonverbal behaviors.

## Introduction

Coding nonverbal behavior using thin slices is a common measurement technique. Thin slices refer to video or audio excerpts extracted from a longer behavioral stream, typically a recorded interaction (Ambady et al., 2000). The shorter excerpts are then coded for specific behaviors or characteristics of individuals in the interaction. Many disciplines use thin slices to capture behavior as a representative measure of that behavior displayed across a longer social interaction including research on: close relationships (e.g., maternal warmth from 5-minute slices; Cheung & Delany, 2022); development (e.g., children’s personality from 1-minute slices; Whalen et al., 2020); interpersonal attraction (vocal attractiveness from 15-second slices; Berry, 1990); organizational contexts (e.g., loudness during decision-making from 1-minute slices; Locke & Anderson, 2015); and clinical diagnostics (facial expressiveness of individuals with autism from 2-minute slices; Frost et al., 2020). Additionally, thin slices are very frequently used for measuring accuracy in judging affective, social, and personality characteristics (Letzring & Funder, 2018; Murphy & Hall, 2021). While previous studies indicate that, for some behaviors, thin-slice measurement is reliable and valid, there remain gaps in the literature in establishing whether different thin-slice measurement metrics (e.g., duration, frequency, rating) may (or may not) be comparable.

Ambady and Rosenthal (1992) coined the term “thin slices” in their meta-analysis on the ability of information contained in brief excerpts to predict other characteristics of target people. Yet, the technique itself of using thin slices for behavioral measurement dates back decades. Olson (1930) reliably measured students’ nervous behaviors (e.g., grimacing) during 5-minute classroom observation periods. Goodenough (1928) measured smiling and laughter, among other behaviors, in 10-second slices across 1-minute observation periods. Nonverbal cues to depression were examined from 2-minute videos of psychiatric patients (Waxer, 1974). Two-minute excerpts of interviews with either lying or honest targets (i.e., individuals whose behavior was being judged) were used to assess nonverbal behaviors of the face and body associated with personality judgments (Ekman et al., 1980). The continued popularity of thin slices as a research paradigm and measurement technique is a testament to their versatility and utility in the behavioral sciences, as well as evidence to their validity.

Researchers are often drawn to thin slices because behavioral measurement is time- and labor-intensive for human coders, typically requiring hours of training before measurement can begin. For comparison, the Facial Action Coding System, a widely-used measure of facial movement, requires 50–100 h of training before coding can begin (Rosenberg & Ekman, 2020). Other popular coding schemes such as the Marital Interaction Coding System (MICS; Weiss & Summers, 1983) and the Motivational Interviewing Skills Code scheme (MISC; Miller & Rollnick, 2002) involve weeks of training with multiple coders. Even simple behavioral measurement, such as counting the frequency of smiles, can involve many hours of human labor (Burgoon & Baesler, 1991; Murphy, 2005). Yet, the advantages of thin slices go beyond their convenience. Researchers may have confidence in this approach as thin slices rely upon the theoretical foundations of behavioral consistency in social interactions and human coders are shown to capture behavioral constructs reliably and validly (Funder & Colvin, 1991; Murphy, 2005; Murphy et al., 2015, 2019).

### Convergent Validity of Coding Metrics

Although there are various ways to quantify behaviors, researchers often utilize metrics of durations, frequencies, and/or ratings. The choice regarding which coding metric to employ is often left up to a researcher’s discretion (Blanch-Hartigan et al., 2018). For instance, in a study of personality as displayed in social interactions, smiles were coded using frequencies as a metric whereas frowns were coded using duration (Gifford, 1994). Given that researchers employ different metrics for the same behavioral measures, often without a theoretical or empirical statement as to why, this begs the question of whether coding metrics are interchangeable. That is, is there convergent validity between coding metrics for the same behavior?

Convergent validity is fundamental to psychological measurement and scale construction (Craig, 2007; John & Benet-Martínez, 2014). To ensure that a psychological measure is valid, one measure should correlate with another measure of the same construct. For instance, self-judgments of personality are often compared to ratings made by knowledgeable acquaintances to establish their validity (e.g., Fast & Funder, 2008) or comparisons between scales to establish new measures of clinical conditions (e.g., Merrell, 1995). Correspondingly, establishing convergent validity for behavioral measurement is important to ensure that measurement metrics are assessing the same underlying construct (i.e., behavior).^1^ Yet, “many measurement decisions in the nonverbal arena are governed more by happenstance, history, or cost than by traditional measurement criteria” (Burgoon & Baesler, 1991, p. 57), a statement that remains as true today as it was decades ago. Despite both the widespread use of thin-slice behavioral coding and the importance of convergent validity in measurement, relatively sparse and sporadic research exists on whether thin-slice coding metrics are comparable.

Burgoon and Baesler (1991) conducted one of the more extensive investigations of convergent validity of nonverbal measurement across 20 vocal and movement-related behaviors.^2^ They compared two coding metrics: molar measurement (global or general judgements of behavior such as rating silences as none, some, and many) and micro measurement (typically single, discrete behaviors such as counting the number of silences longer than 1 s). While 13 of the 20 behaviors showed convergent validity with effect sizes of *r* >.30, the authors were careful to note that concurrent validity was dependent on the specific behavior being measured.

Other studies specifically comparing coding metrics include Friesen et al. (1979), who compared coding hand gestures with both duration and frequency metrics. The authors concluded that the coding metrics were so highly correlated that they could be interchangeable. Another study compared rating scales and behavior counts in preschool children’s dominance and dependency behaviors, and the convergent validity correlations between metrics ranged from .34 to .65, depending on number of coders and observation periods (Moskowitz & Schwarz, 1982). Fujiwara and Daibo (2014) compared the coding of gestures, adaptors (defined as self-touching), and utterances as measured with rating scales and event recording (presence/absence of behavior every 30 s). Results indicated strong convergent validity correlations ranging from .62 to .86. While these studies provide some promising evidence of convergent validity in coding metrics, there is little systematic research into thin-slice coding metrics for commonly measured nonverbal behaviors such as smiling and eye gaze when using human coders. Our project builds on and extends this previous literature using a systematic approach across multiple datasets and dyadic settings.

### Overview of Current Project

The purpose of the present research is to bridge the gap between the value of using thin slices for behavioral coding and lack of empirical evidence to support selecting one measurement metric over another. We investigated the convergent validity of thin-slice coding metrics to potentially provide empirical data to aid researchers making coding decisions. To do so, we gathered five pre-existing video datasets of dyadic interactions (see dataset inclusion criteria detailed in Method). Four behaviors (gaze, gestures, nods, smiles) were selected for analyses. Selection of these four behaviors was based on earlier studies indicating that these behaviors can be reliably measured from thin-slice extracts and adequately represent behavioral displays of longer interactions (Murphy, 2005; Murphy et al., 2015, 2019). We also chose these behaviors given their popularity across multiple psychological domains.

Three coding metrics (duration, frequency, rating) were selected based on typical measurement scales used by researchers for coding the four behaviors with human coders:^3^ amount of eye gaze (duration; e.g., Gifford & O’Connor, 1987; Murphy et al., 2016) and gaze ratings (e.g., Hampton et al., 2019; Pansa-Henderson & Jones, 1982; Winstead et al., 1992); number of gestures (frequency; e.g., Friesen et al., 1979; Leikas et al., 2012; Nicoladis et al., 2022; Pınar et al., 2021) and gesture ratings (e.g., Borkenau & Liebler, 1995; Takahashi et al., 2010); number of nods (frequency; e.g., Ambady et al., 2002; DeSteno et al., 2012; Noller & Callan, 1989) and nod ratings (e.g., Wall et al., 2018); time spent smiling (duration; e.g., Frank et al., 1993; Papa & Bonanno, 2008), number of smiles (frequency: e.g., Hamel et al., 2021; Murphy, 2005; Shrout & Fiske, 1981), and smile ratings (e.g., Katsikitis et al., 1990; Messinger et al., 2008).

We predicted that coding metrics would be positively correlated with one another in line with previous findings (Burgoon & Baesler, 1991; Friesen et al., 1979; Fujiwara & Daibo, 2014). We had no formal predictions about the magnitude of correlations, potential differences in effect size magnitude between coding metrics, or potential differences between behaviors, so we conducted exploratory analyses to answer the following research questions:

**RQ1** Are there differences in effect size magnitude between coding metrics?

**RQ2** Do any potential differences in effect size magnitude depend on specific behaviors?

## Method

Data are available on the Open Science Framework (https://osf.io/d35rw/) and the project was pre-registered (https://aspredicted.org/b2723.pdf).^4^

### Dataset Inclusion Criteria

All datasets were from previous studies originally designed and conducted for other purposes. As such, the selection of the datasets was non-random and selected simply based on convenience and availability. At the same time, the original studies were conducted for unrelated purposes and no convergent validity analyses were conducted prior to the current project development or selection for inclusion. Some of the video datasets were previously coded for the purposes of the original study, and some of the previously coded data were eligible for use in the present study (indicated as such in following study descriptions). Regardless, all video datasets included were coded further for the purposes of the present study. Target stimuli contained within each video dataset were collected with approval by corresponding institutional review boards in accordance with the APA Ethical Principles of Psychologists and Code of Conduct (American Psychological Association, 2017) or the European Code of Conduct for Research Integrity (ALLEA, 2023). The total sample size of each video dataset was dependent on the constraints of the original studies. Thus, analyses for the present project had *N*s in the range of 50 to 150 targets per video dataset. Not every behavior was measured in every study, and not every behavior was coded with all metrics, but every behavior was coded with at least two metrics in at least two studies for inclusion, with one exception (see Results for further information).

### Behavior and Coding Metrics Descriptions

The coding manual with complete definitions and descriptions is provided in the open access repository. Reliable raters conducted coding using similar operational definitions of the behavior. There were slight variations in behavior operational definitions related to the purpose and constraints of the original study, but all studies adhered to the general descriptions of behavior as follows: (1) gaze = *eye gaze at interaction partner [by] looking at the interaction partner’s eyes or face*; (2) nods = *series of up-and-down head bobbing movements*, *usually to signal agreement with a speaker*; (3) gestures = *hand and arm movements*, *usually accompanying speech and to punctuate a speech-related point (but not necessarily)*; and (4) smiles = *generally… [representing] activation of the zygomatic major muscles which retract and pull the lip corners upwards resulting in a characteristic “smiling expression.”*

Regarding the three coding metrics, the frequency coding definition was the count of a behavior. Duration coding was the length of time the behavior was displayed and recorded to 1/10th per second. Ratings were made at a molar level (i.e., holistic) as an impression of how much a behavior was displayed as rated on a scale of 1 (*very little or no behavior displayed*) to 9 (*behavior extensively or substantially displayed*).

Across all studies, behaviors were coded by reliable coders. Reliability coefficients are reported as a correlation coefficient (*r*) when coding was done by one coder but reliability was established against a second coder for a portion of the clips, thus describing the reliability of the main coder; or as Cronbach’s alpha (α) when coding was done by two or more coders for all clips, in which case the coefficient expresses the reliability of the group of coders.

### Video Dataset Descriptions

Five studies provided video datasets for behavioral coding. Each study is briefly described below, including which behaviors and metrics were coded from that study.

#### Study 1

##### Target Videotaping Procedure

Targets were university students in a study examining social support interactions between friends (Murphy & Marroquín, 2024); *N* = 89 (51 women, 37 men, 1 no response regarding gender); *M*_age_ = 18.99 years, *SD* = 1.02; 82% in their first or second year of university. 48% reported their ethnicity as Caucasian, 22% Asian or Asian American, 14% Hispanic, 10% Black or African American, and 6% reporting other or not reported.

##### Coding Procedures and Interrater Reliability

Two behaviors were previously coded in thin-slices (gaze duration, smile frequency) and data were obtained from the original study (Murphy & Marroquín, 2024). For the purposes of the present study, additional coding was conducted for gaze ratings and smile duration. All coding data used 2-minute slices after the first minute. Coder reliability was established by correlating their assessments on 10 targets. Coding reliability was as follows: gaze duration *r* = .83, gaze rating *r* = .83, smile duration *r* = .83, and smile frequency *r* = .89. Two women coded smiling frequency, and one woman coded gaze rating, smile duration, and smile rating.

#### Study 2

##### Target Videotaping Procedure

Targets were 139 undergraduate students (71 female, 68 male; *M*_age_ = 18.65 years, *SD* = 1.16), paired in same-gender dyads, engaged in a videotaped negotiation role-play discussion about a job contract (Schlegel, 2021; Schlegel et al., 2017); 47% reported their ethnicity as Caucasian, 27% Asian or Asian American, 10% mixed, 7% Black or African American, 5% Hispanic, 4% reporting other or not reported. One person was randomly assigned the role of the candidate, and the other person played the recruiter. The average duration of the discussions was 12.5 min.

##### Coding Procedure and Interrater Reliability

All coding was completed for the purposes of the present study. Three-minute slices from each target (i.e., both dyad members) after the first minute of the conversation were coded for smile frequency and duration. Some targets could not be coded due to technological difficulties, resulting in varying *N*s in listwise deletion. Coder reliability was established between the assigned coder and another individual by correlating their assessments on 3-minute slices: smile frequency *r* = .93 (19 targets) and smile duration *r* = .88 (18 targets). One woman coded both smiling frequency and duration.

#### Study 3

##### Target Videotaping Procedure

Targets were participants in a study examining the relationship between behavioral synchrony and interpersonal accuracy (Stosic, 2021). Dyads were composed of strangers and given the prompt to identify as many things in common with one another as possible within a 5-minute period while being videotaped. The entire interaction took place over a video conferencing platform. For the purposes of the present study, one participant per dyad was randomly selected for analysis, *N* = 97 (58 women, 37 men, 2 gender diverse or did not identify); *M*_age_ = 20.48 years, *SD* = 3.88. A total of 89 participants identified as White (91.8%), three as African American (3.1%), four as Asian (4.1%), and one as other (1.0%). Additionally, seven (7.2%) identified as Hispanic or LatinX.

##### Coding Procedures and Interrater Reliability

Two-minute slices from each participant beginning after the first minute of the 5-minute discussion were coded for gaze (rating, duration), nods (frequency, rating), and smiles (frequency, rating, duration).

All coding was completed for the purposes of the present study. Five coders were trained to complete ratings on all behaviors (all coders coded all of the behaviors). All coders who completed ratings identified as women. For behaviors coded using a rating approach, the reliabilities were as follows: gaze *α* = .82, nod *α* = .84, and smiles *α* = .92.

Two new coders completed frequency and duration coding on the following behaviors: gaze duration, nod frequency, and smiling frequency. Both coders coded all of the behavior clips. One of these coders identified as a man and one as a woman. The reliabilities were: gaze duration *α* = .83, nod frequency *α* = .87, and smile frequency *α* = 0.80.

Finally, eleven new coders completed duration coding for smiling only. Of these coders, six identified as women, one as a man, and one as a nonbinary person, with the remaining three choosing not to self-identify. All coders coded all of the behavior clips. For smiling duration the reliability was *α* = .97.

#### Study 4

##### Target Videotaping Procedure

Targets (*N* = 147, all men, *M*_age_ = 22.58 years, *SD* = 3.71) were participants in a study examining the effect of job interview methods on participants’ perception of interview behaviors (Kleinlogel et al., 2023). The targets participated in a 10-minute job interview in one of the three experimental conditions: (1) human-agent interface (targets interviewed by a virtual recruiter), (2) human-machine interface (targets interviewed through written questions), and (3) face-to-face (targets interviewed by a recruiter) while being video recorded. Targets were instructed to act as they would during a real job interview for the selected job position.

##### Coding Procedures and Interrater Reliability

All coding was completed for the purposes of the present study. From targets’ 10-minute discussion, 2-minute slices (beginning after the first minute of discussion) were coded for gesture (frequency, rating) and nods (frequency, rating). Reliability was established between a pair of coders by correlating the coding of 2-minute slices from 10 targets: gesture frequency *r* = .99, gesture rating *r* = .93, nod frequency *r* = .97, and nod rating *r* = .90. One coder measured gesture and nod frequency, and another coder measured gesture and nod ratings. All coders identified as women.

#### Study 5

##### Target Videotaping Procedure

Targets (*N* = 70, all men, *M*_age_ = 21.61 years, *SD* = 3.47) were participants in a study investigating the effect of self-observation on participants’ anxiety and confidence during a presentation (Renier et al., 2025). Targets were instructed to present themselves to a virtual audience (i.e., 10 virtual humans) with the goal of making the best possible impression. The experimenter told the participants that a panel of judges would view their video presentation and rate their performance. The experimenter also informed them that, due to sanitary conditions, they would be required to perform public self-presentations while wearing a mask. As a result, the experimenter highly recommended that they avoid being expressive in the face and be expressive in their gestures and movements.

##### Coding Procedures and Interrater Reliability

All coding was completed for the purposes of the present study. From targets’ 3-minute presentation, 2-minute slices (beginning after the first minute) were coded for gesture (frequency, rating) and nods (frequency, rating). Studies 4 and 5 measured the same behaviors with the same metrics and used the same coders; therefore, Study 4 reliability information applies to Study 5.

## Results

Convergent validity was calculated by correlating one coding metric with a second coding metric for a single behavior within a given study. For example, in Study 1, gaze was coded with both the duration metric and the rating metric. These two metrics were then correlated; the resulting correlation (*r* = .81) represents convergent validity between duration and rating metrics for Study 1 gaze behavior. Though there does not seem to be established standards on what constitutes an acceptable value to establish convergent validity, an *r* ≥.50 would indicate at least 25% shared variance between the two variables, indicating that the variables are at least moderately related. Complete results are in Table 1. (Means and *SDs* for coding metrics and behaviors measured within each individual study are provided in supplementary materials.)

As shown in Table 1, strong convergent validity was found for each metric comparison within each behavior between studies (*r*s ranging from .51 to .85) with one exception: the convergent validity for nod frequency compared with nod rating was *r* = .30 in Study 5. Correlations were then combined across studies in a meta-analytic fashion to determine an estimated average effect for the convergent validity of the two measurement metrics for a given behavior (Goh et al., 2016). Convergent validity was strong across metric comparisons within each behavior ($\overset{‾}{r}s$ ranging from .58 to .82), with the lowest value for the comparison of nod frequency coding to nod rating coding $\left(\overset{‾}{r}=.58\right)$.^5^ Although only one study investigated smiling using both frequency and rating metrics and was thus ineligible for meta-analytic combination, the results still aligned with the other findings in showing strong convergent validity (*r* = .85) for that comparison. These results confirm our hypothesis that coding metrics would be positively correlated with one another for each behavior.^6^

To address our two exploratory research questions (RQ1 and RQ2) regarding similarities or differences in the convergent validity magnitude between coding metrics or specific behaviors between studies, we tested whether there were statistically significant differences between correlations for each coding metric comparison within a behavior. This calculation determines if the effect size magnitudes were statistically significantly different between studies (Soper, 2024; but also, see footnote 5). Only two statistically significant differences were found. Regarding the comparison for nod frequency with nod rating, Study 3 convergent validity of *r* = .80 was significantly higher than both Study 4 (*r* = .51) and Study 5 (*r* = .30) convergent validity (*p*s ≤ .05). These results suggest some degree of variation in the magnitude of convergent validity between studies specifically for nod frequency and rating metrics.

## Discussion

Aligning with Burgoon and Baeler’s (1991) earlier observations about “the need for more empirical examination of nonverbal measurement processes” (p. 76), we investigated the convergent validity of thin-slice coding metrics for four commonly measured nonverbal behaviors (gaze, gestures, nods, smiles). Using five existing video datasets, these four behaviors were coded using different coding metrics (frequency, duration, and rating). The results supported our hypothesis that different thin-slice coding metrics for a given behavior would be positively correlated. Yet, beyond the positive correlations, the effect sizes were quite strong, as indicated by the meta-analytic results as presented in the right-most column of Table 1. The range of $\overset{‾}{r}s$ (from .58 to .82), indicates 34–67% of shared variance between coding metrics for a given behavior.

Our additional research questions asked whether studies comparing the same metrics for the same behavior would show similarities or differences in the convergent validity effect size magnitude. We found only one instance of a statistically significant difference between studies measuring the same behavior with the same two metrics. For the three studies comparing the measurement of nods by frequency and rating, Study 3 convergent validity was significantly higher in comparison to Studies 4 and 5. These differences suggest that there can be variation in the convergent validity of frequency coding with ratings specifically for nods, though the overall meta-analytic average ($\overset{‾}{r}=.58$) still provides strong evidence for convergent validity and support that these behaviors are likely capturing a shared underlying construct. Importantly, demonstrating that there are statistically significant differences simply indicates variation that could be due to particular qualities of each study’s design, but does not indicate a lack of convergent validity. Additionally, no comparisons involving gaze, gestures, or smiles showed statistically significant differences between studies.

To be clear, not every metric comparison was conducted on every behavior. Rather the results present a snapshot of convergent validity for thin-slice coding metrics for a given behavior. There were several comparisons that were not coded or compared: duration by frequency for gaze, gesture, or nods; duration by frequency for gestures and nods; and frequency by rating for gaze. Although a potential weakness of the project, we feel confident that these results, in addition to previous research in the area, provide assurances that selected coding metrics for some nonverbal behaviors may be fairly interchangeable. Additionally, stimuli from each dataset were originally collected independently and for entirely different purposes than testing behavioral measurement convergent validity; as such, the results are not limited to a single dataset or study. At the very least, these data provide solid empirical evidence on which researchers may base future coding choices, when using thin-slice behavioral coding for these behaviors and metrics.

There still may be reasons to select one metric over another. For instance, there may be a conceptual difference between simply counting the number of smiles, which is a micro-level assessment, compared to rating smiling on a Likert scale, which may capture how “smiley” someone appears at a macro-level and thus may integrate the coder’s impression of duration, intensity, and frequency. Researchers may prioritize certain metrics based on the context of their study, the nature of their research questions, or the constraints of available resources. For example, frequency coding may be preferred in studies aimed at capturing precise, moment-to-moment behavioral changes, whereas rating scales might be more suitable for holistic impressions of behavior across time. The results of the present set of studies, however, suggest that ratings of behaviors, which are substantially less labor-intensive to collect, are often valid proxies for micro-level assessments of behavior.

Although utilizing ratings as a metric is more efficient than measuring behaviors by their frequency and duration, it can still take human coders quite some time and requires more than one human coder to achieve reliability. Thus, human coding is time-consuming, labor-intensive, and subject to variability in interpretation. Even with extensive training and inter-rater reliability checks, cognitive fatigue and subjective bias can influence coding decisions.

Automated systems, which often rely on machine learning algorithms, offer an alternative method to human coding of thin slices that is less time- and labor-intensive. These systems are trained to be sensitive to “micro” behaviors, such as those employed in the current study, and are able to quantify behaviors using metrics such as frequency, duration, or intensity. Automated coding systems offer consistency, speed, and scalability. They can process large datasets of videos in a fraction of the time it would take a human team and are not affected by fatigue. However, automated coding systems can reflect and perpetuate biases present in their training data, which is often out of the hands of the researcher by the time they are using the platform (e.g., Noldus’ FaceReader). Such systems may struggle with subtle context, misinterpret ambiguous behavior, and lack the capacity for human intuition or moral reasoning. Additionally, unless a researcher has developed their own machine learning program, there is a “black box” problem wherein it is difficult to fully understand how decisions are made, which can be problematic for interpretation.

Although some research has begun to compare these automated systems with human coders (e.g., Fujiwara et al., 2021; Novotny & Bente, 2022; Skiendziel et al., 2019; Stosic et al., 2025), this area of research is quite new in comparison to the history of thin-slice coding utilizing human coders and still requires further understanding of the gains and losses in reliability and validity when selecting one measurement tool over another. Thus, future research should continue to establish convergent validity across coding metrics between human coders and automated systems, especially as advances in artificial intelligence continue to impact the field of psychology and nonverbal communication. We believe that, together with human coders, artificial systems can augment analyses of human behavior, but must be done in conjunction with human oversight due to the biases inherent in both methods.

### Limitations and Future Directions

Limitations to these findings include the small number of behaviors examined. While four of the most commonly measured nonverbal behaviors were selected, there are numerous other nonverbal behaviors that may not show the same patterns. These were also “micro” behaviors, as compared to molar constructs (e.g., friendly, self-assured), which are more holistic in nature. Whether convergent validity between coding metrics extends to other behaviors or constructs remains unknown.

As previously mentioned, there does not appear to be an agreed-upon numeric standard for establishing convergent validity. In our results, the lowest mean *r* of .58 (for comparing nods measured by frequency to rating) indicates 34% shared variance between those metrics for measuring nods. Whether this represents sufficient evidence to establish convergent validity is left up to the reader, though our findings do support and align with previous thin-slice convergent validity research, which concluded validity was established with values between .34 and .86 (Fujiwara & Daibo, 2014; Moskowitz & Schwarz, 1982). We also acknowledge limitations based on our sample populations and convenience sample of datasets, given that study participants were primarily university students, which potentially restricts generalizability and representativeness of our findings.

While our results provide some evidence of convergent validity between coding metrics of the same behavior, there remains the question as to whether there is equivalent predictive validity of outcome variables with different coding metrics. Predictive validity of thin slices refers to how well a behavioral thin slice “predicts” (i.e., correlates) with an outcome variable (Murphy & Hall, 2021). For instance, how strongly does a thin slice of smiling predict participants’ friendliness during an interaction? Previous research indicates 1-min slices of specific nonverbal behaviors, such as nods and self-touch, demonstrated adequate predictive validity for a variety of outcomes such as making a good impression and dominance (Murphy et al., 2019). Yet, questions remain as to whether the measurement metric of the thin-slice behavior differs depending on how a behavior is coded. That is, does the predictive validity of smiling to friendliness differ if smiling is measured with a rating or as frequency? The current research is unable to answer such questions, but does present promising future directions in investigating the validity of thin-slice behavioral metrics.

## Conclusion

Across five studies, we provide evidence for the convergent validity of thin-slice coding metrics for four commonly studied nonverbal behaviors: gaze, gestures, nods, and smiles. The strength of the correlations observed between different metrics supports the notion that, in many cases, these approaches may be used interchangeably without substantial loss of measurement integrity. In other words, researchers without a-priori guidance as to which coding method to select may feel at ease in knowing that less labor-intensive methods, such as ratings, are strongly convergent with more labor-intensive methods such as coding for frequency or duration. However, the observed variability in effect sizes for certain behaviormetric pairings highlights the importance of context-specific decision-making when selecting coding methods. For instance, counting nods as each individual head bob could differ from counting nods as “series of up-and-down head bobbing movements,” which was done for this project. Readers of thin-slice behavioral research also benefit from these findings as they can have confidence in the convergent validity of coding metrics, potentially aiding their interpretation and application of the research. These findings offer practical guidance, encouraging researchers to consider the unique affordances of each metric relative to their research goals.

## Supplementary Material

supplementary material

**Supplementary Information** The online version contains supplementary material available at https://doi.org/10.1007/s10919-025-00489-w.

## Figures and Tables

**Table 1:** Convergent validity between coding metrics within behavior and mean weighted effect size estimates across studies

Behavior and Coding Metric	*N*	Study correlation	95% CI	Mean weighted *r* [95%CI]

Gaze				
Duration with Rating	89	Study 1 *r*=.81	[.72, .87]	
Duration with Rating	97	Study 3 *r*=.84	[.77, .89]	.82 [.77, .86]**
Gestures				
Frequency with Rating	124	Study 4 *r*=.72	[.62, .80]	
Frequency with Rating	70	Study 5 *r*=.62	[.45, .75]	.68 [.58, .11]**
Nods				
Frequency with Rating	97	Study 3 *r*=.80_a_	[.71, .86]	
Frequency with Rating	124	Study 4 *r*=.51_b_	[.37, .63]	
Frequency with Rating	70	Study 5 *r*=.30_b_	[.07, .50]	.58 [.22, .80]**
Smiles				
Frequency with Duration	86	Study 1 *r*=.75	[.64, .83]	
Frequency with Duration	139	Study 2 *r*=.65	[.54, .74]	
Frequency with Duration	97	Study 3 *r*=.81	[.73, .87]	.74 [.47, .88]**
Frequency with Rating	97	Study 3 *r*=.85	[.78, .90]	

Note: Correlations with different subscripts within metric behavior comparisons differ at the *p*≤05 level. Mean weighted *r* column calculations via Comprehensive Meta Analysis v. 4.0 (Borenstein et al., 2021); statistical significance difference calculations via Soper (2024)

** *p*<.01

## Data Availability

Data are available on the Open Science Framework https://osf.io/d35rw/. The project was pre-registered at https://aspredicted.org/b2723.pdf.

## Footnotes

1 To be clear, comparing metrics is distinct from establishing reliability among coders. Researchers typically assess human coder reliability (i.e., inter-rater reliability) by comparing two or more coders’ measurements who are using the same metric. Once reliability is established, the human coder functions as a reliable measurement “tool” to assess the given construct. Reliability establishes the consistency between coders whereas convergent validity refers to the consistency between different coding metrics. As with establishing the reliability and validity of any measurement tool, reliability must be established before validity can be assessed.

2 Burgoon and Baesler (1991) used the term concurrent validity, which implies that one of the measurement metrics serves as the existing ‘gold standard’ against which to compare the second metric. We believe the comparison of coding metrics is a form of convergent validity, which is used to demonstrate that one measure of a construct aligns with a second measure of the same construct.

3 Nonverbal behavior may be measured using tools other than human coding, such as eye-tracking for gaze duration (e.g., Melnyk et al., 2014), speaking time using automated methods (e.g., Frauendorfer et al., 2014), or smiling using software systems (e.g., FaceReader; www.noldus.com/facereader). Automated measurement can reduce the human labor costs but nonverbal researchers across disciplines continue to use human coding for a variety of reasons including the limits to which constructs are able to be captured via automated methods and access to the technology and technological skills needed to use automated techniques (Murphy & Hall, 2021).

4 The authors acknowledge that the preregistration included a second series of analyses involving predictive validity, which examines how well a behavior predicts an outcome variable. The planned analyses intended to examine whether different coding metrics would show differences in the levels of predictive validity for a given outcome variable (e.g., does the magnitude of the relationship between smiling and an outcome variable such as extraversion change depending on whether smiling is coded as a frequency or as a rating?). In previous predictive validity research, the inclusion criteria maintained that a thin-slice behavior (coded at 1–2 min) had to be significantly correlated with an outcome variable for inclusion in further analysis (Murphy & Hall, 2021; Murphy et al., 2019). In the current datasets, we found very few (and very small) significant correlations between most thin-slice behaviors and outcome variables. Because the purpose of the present work aimed to synthesize across multiple datasets and provide guidance to researchers on which metrics to select, we opted not to conduct the planned analyses, as it was clear the results would yield little informative data on which metrics to select in predictive validity designs.

5 All mean weighted *r*s were statistically significant, though statistical significance is of less importance in validity estimation because validity is established based on the magnitude of effects rather than statistical significance. This also accounts for the lack of any *a prior*i power analysis.
