# Supplementary material for: How Long? How Many? How Much? Evidence of Convergent Validity Among Thin-Slice Behavioral Coding Metrics
Source: J Nonverbal Behav. Author manuscript; Available in PMC 2026 Mar 31. (PMC13035377; doi:10.1007/s10919-025-00489-w)
Supplement: supplementary material [file NIHMS2147547-supplement-supplementary_material.docx]

**Supplementary Material**

**Supplementary Table**

*Descriptive Statistics of Measured Behaviors by Study*

|  |  | Study 1  *N* = 89 | Study 2  *N* = 139 | Study 3  *N* = 97 | Study 4  *N* = 147 | Study 5  *N* = 70 |
| --- | --- | --- | --- | --- | --- | --- |
|  |  |  |  |  |  |  |
|  |  | *M* (*SD*) | *M* (*SD*) | *M* (*SD*) | *M* (*SD*) | *M* (*SD*) |
| Gaze | |  |  |  |  |  |
|  | Duration | 97.96 (16.28) | -- | 92.38 (5.89) | -- | -- |
|  | Rating | 6.97 (1.88) | -- | 5.89 (1.22) | -- | -- |
|  |  |  |  |  |  |  |
| Gesture | |  |  |  |  |  |
|  | Frequency | -- | -- | -- | 4.72 (4.43) | 10.77 (7.65) |
|  | Ratings | -- | -- | -- | 2.50 (2.19)^a^ | 3.79 (2.60) |
|  |  |  |  |  |  |  |
| Nods | |  |  |  |  |  |
|  | Frequency | -- | -- | 6.39 (3.19) | 7.56 (4.85) | 4.34 (4.45) |
|  | Ratings | -- | -- | 3.63 (1.25) | 3.19 (2.09)^a^ | 2.51(1.97) |
|  |  |  |  |  |  |  |
| Smiles | |  |  |  |  |  |
|  | Duration | 33.13 (25.95) | 30.14 (29.37) | 41.81 (20.11) | -- | -- |
|  | Frequency | 4.49 (3.25) | 5.60 (3.31) | 5.82 (2.81) | -- | -- |
|  | Rating | -- | -- | 4.78 (1.86) | -- | -- |

*Note*. Study 3 coding based on 3-min slices, all other studies based on 2-min slices. Duration reported in seconds, ratings reported on a scale of 1 – 9. “- -" = not measured.

^a^ *N* = 124
